# Interventions to Disrupt Coronavirus Disease Transmission at a University, Wisconsin, USA, August–October 2020

**DOI:** 10.3201/eid2711.211306

**Published:** 2021-11

**Authors:** Dustin W. Currie, Gage K. Moreno, Miranda J. Delahoy, Ian W. Pray, Amanda Jovaag, Katarina M. Braun, Devlin Cole, Todd Shechter, Geroncio C. Fajardo, Carol Griggs, Brian S. Yandell, Steve Goldstein, Dena Bushman, Hannah E. Segaloff, G. Patrick Kelly, Collin Pitts, Christine Lee, Katarina M. Grande, Amanda Kita-Yarbro, Brittany Grogan, Sara Mader, Jake Baggott, Allen C. Bateman, Ryan P. Westergaard, Jacqueline E. Tate, Thomas C. Friedrich, Hannah L. Kirking, David H. O’Connor, Marie E. Killerby

**Affiliations:** Centers for Disease Control and Prevention, Atlanta, Georgia, USA (D.W. Currie, M.J. Delahoy, I.W. Pray, G.C. Fajardo, D. Bushman, H.E. Segaloff, C. Lee, J.E. Tate, H.L. Kirking, M.E. Killerby);; University of Wisconsin–Madison, Madison, Wisconsin, USA (G.K. Moreno, A. Jovaag, K.M. Braun, D. Cole, T. Shechter, C. Griggs, B.S. Yandell, S. Goldstein, P. Kelly, C. Pitts, J. Baggott, R.P. Westergaard, T.C. Friedrich, D.H. O’Connor);; Wisconsin Department of Health Services, Madison (I.W. Pray, D. Cole, H.E. Segaloff, R.P. Westergaard);; Public Health Madison and Dane County, Madison (K.M. Grande^,^ A. Kita-Yarbro, B. Grogan, S. Mader);; Wisconsin State Laboratory of Hygiene, Madison (A.C. Bateman)

**Keywords:** COVID-19, SARS-CoV-2, coronavirus disease, severe acute respiratory syndrome coronavirus 2, viruses, respiratory infections, zoonoses, students, university, dormitory, Wisconsin, University of Wisconsin–Madison, whole-genome sequencing, COVID-19 testing, United States

## Abstract

University settings have demonstrated potential for coronavirus disease (COVID-19) outbreaks; they combine congregate living, substantial social activity, and a young population predisposed to mild illness. Using genomic and epidemiologic data, we describe a COVID-19 outbreak at the University of Wisconsin–Madison, Madison, Wisconsin, USA. During August–October 2020, a total of 3,485 students, including 856/6,162 students living in dormitories, tested positive. Case counts began rising during move-in week, August 25–31, 2020, then rose rapidly during September 1–11, 2020. The university initiated multiple prevention efforts, including quarantining 2 dormitories; a subsequent decline in cases was observed. Genomic surveillance of cases from Dane County, in which the university is located, did not find evidence of transmission from a large cluster of cases in the 2 quarantined dorms during the outbreak. Coordinated implementation of prevention measures can reduce COVID-19 spread in university settings and may limit spillover to the surrounding community.

Severe acute respiratory syndrome coronavirus 2 (SARS-CoV-2), which causes coronavirus disease (COVID-19), can spread rapidly within congregate settings, including institutions of higher education (IHEs) (*1*,*2*). During August–December 2020, as IHEs around the United States resumed in-person instruction, IHE-associated SARS-CoV-2 cases began to rise (*3*). By February 2021, >530,000 COVID-19 cases linked to US IHEs had been identified (*4*). In many IHE settings populated substantially by young adults 18–24 years of age (*5*), susceptibility to severe COVID-19 is lower than for older populations (>65 years of age) (*6*). Adhering to physical distancing is also challenging for young adults, for whom interaction with peers and social networks is important (*7*).

As students returned to in-person learning, high-density clustering within on-campus housing may have increased transmission and resulted in community outbreaks (*8*–*10*; M.S. Andersen, et al., unpub. data, https://doi.org/10.1101/2020.09.22.20196048; C.S. Richmond, et al., unpub. data, https://doi.org/10.1101/2020.10.12.20210294). One study using whole-genome sequencing (WGS) data, which can be used to track specific SARS-CoV-2 lineages through space and time (*11*–*16*; M. Zeller et al., unpub. data, https://doi.org/10.1101/2021.02.05.21251235), suggested that SARS-CoV-2 transmission chains beginning or proliferating on IHE campuses may lead to spread within the surrounding community, including to populations at higher risk for severe disease (C.S. Richmond, et al., unpub. data). Therefore, strategies to prevent SARS-CoV-2 spread on IHE campuses and between IHEs and the community are needed.

We used epidemiologic and genomic data to describe an outbreak of SARS-CoV-2 infection at the University of Wisconsin–Madison (UW-Madison; Madison, WI, USA) shortly after its reopening for the fall 2020 semester. We report the trajectory of the outbreak and describe measures taken to reduce transmission. In addition, using genomic data, we investigated whether SARS-CoV-2 lineages associated with outbreaks at dormitories may have spread into the community surrounding UW-Madison.

The Western Institutional Review Board obtained a waiver of Health Insurance Portability and Accountability Act authorization (WIRB #1-1290953-1) to obtain the clinical specimens for whole-genome sequencing. Our analysis was reviewed by Centers for Disease Control and Prevention (CDC) and was conducted consistent with applicable federal law and CDC policy (45 C.F.R. part 46.102(l)(2), 21 C.F.R. part 56; 42 U.S.C. Sect. 241(d); 5 U.S.C. Sect. 552a; 44 U.S.C. Sect. 3501 et seq.). The Institutional Review Board at UW-Madison determined these activities were nonresearch public health surveillance.

## Methods

### Setting

UW-Madison is a large public university in the midwestern United States; during the fall 2020 semester, the university had ≈45,540 enrolled students and 23,917 staff (*17*). UW-Madison offered a combination of in-person and virtual classes for this semester. Undergraduate students living in on-campus dormitories and moved in on preassigned days during August 25–31, 2020. They were tested for SARS-CoV-2 on move-in day and subsequently required to undergo testing every 2 weeks regardless of symptoms. Appointment-based testing for all students and staff was also available free of charge. Testing was conducted on anterior nasal swab specimens using real-time reverse transcription PCR (rRT-PCR) tests authorized by the Food and Drug Administration. UW-Madison instituted a mandatory COVID-19 student pledge at the start of the semester, which required mask usage at all times (except within students’ own rooms), physical distancing when possible, self-monitoring for symptoms, and limited gatherings in accordance with local public health guidelines (*18*). Students were provided a symptom screening tool for symptom self-monitoring; those screening positive were instructed to schedule a test and self-isolate (except for medical care) until receiving a negative result.

Isolation facilities were established in designated dormitories to temporarily house students living on-campus who tested positive for SARS-CoV-2. Students living on campus who were identified as close contacts of persons testing positive for SARS-CoV-2 (defined as being within 6 feet of an infected person for >15 minutes within a 24-hour period from 2 days before illness onset or positive specimen collection through the end of isolation) were quarantined in individual single rooms in local hotels for 14 days; meals were delivered to the rooms, and students were tested for SARS-CoV-2 during the first and second week of quarantine. If a quarantined student tested positive, they isolated in the same quarantine location. Nonquarantined students who tested positive were transferred to designated isolation dormitories. Isolation lasted for 10 days after symptom onset, or 10 days after positive specimen collection for those who were asymptomatic, consistent with CDC recommendations (*19*).

As the semester progressed, some modifications to the quarantine procedure were required. Given the high frequency of positivity within 2 dormitories (dorms A and B) during universal testing events, all students living in these 2 dormitories were asked to quarantine within their hall for 2 weeks to mitigate transmission. During the dormitory quarantine period, students were asked to wear a face covering when leaving their room, refrain from congregating, self-monitor for symptoms, test onsite, and stay in their dormitory. Residents testing positive were moved to an isolation facility, and roommates of residents testing positive initially quarantined within their dormitory room. Approximately 1 week into the dormitory quarantine, roommates of those with positive cases were moved to alternative quarantine facilities. Students could also choose to quarantine at their permanent home; those students could return to the dormitory after the quarantine ended and they provided a negative test result.

County-level ordinances passed earlier in the summer also applied to the UW-Madison community. As of July 13, 2020, emergency order no. 8 from Dane County, which includes Madison, mandated the use of face coverings when in public, limited the size of public gatherings, limited restaurant capacity, and closed bars except for takeout and socially distanced outdoor seating (*20*).

### Epidemiologic Data Analysis

We used Wisconsin Electronic Disease Surveillance System (WEDSS) data to describe demographic characteristics, location of on-campus clusters, and symptoms of COVID-19 cases. We defined a UW-Madison–affiliated SARS-CoV-2 infection as a positive rRT-PCR test result in a specimen collected from a UW-Madison student or staff member during August 1–October 31, 2020. We calculated daily percent positivity (defined as the number positive SARS-CoV-2 specimens collected on a given day divided by the total number of specimens collected) and attack rates within 19 dormitories (occupancy range 26–1,195 residents) using campus testing program data. We merged campus testing data with data from the University Housing department to determine housing location of students living on-campus as of September 22, 2020. We defined index cases for roommate attack rate calculations as the resident with the first positive SARS-CoV-2 test result within a room in a dormitory. We defined roommate attack rate as the proportion of susceptible students (roommates of an index case that had not previously tested positive for SARS-CoV-2) who had a positive SARS-CoV-2 specimen collected 2–14 days after the index case specimen collection. We performed epidemiologic data analyses using SAS software version 9.4 (SAS Institute, https://www.sas.com), and RStudio version 1.2.1335 (RStudio Team, https://www.rstudio.com).

### Whole-Genome Sequencing

Sequences for this investigation were derived from 262 anterior nasal swab samples collected during September 8–22, 2020, from UW-Madison students living in dormitories A and B. We selected these samples for sequencing because they were the largest outbreaks among students living in on-campus housing; we sought to determine whether the outbreaks between the 2 dormitories were distinct. We extracted viral RNA from 100 μL of viral transport medium by using the Viral Total Nucleic Acid Purification kit (Promega, https://www.promega.com) on a Maxwell RSC 48 (Promega) instrument and eluted it in 50 μL of nuclease-free H_2_O. We synthesized cDNA using a modified ARTIC Network approach (*21**–**23*). In brief, we reverse transcribed 11 µL of virual RNA with SuperScript IV Reverse transcription (Invitrogen, https://www.thermofisher.com) according to the manufacturer’s guidelines. We used ARTIC version 3 primers (IDT, https://www.idtdna.com/pages/landing/coronavirus-research-reagents/ngs-assays) for SARS-CoV-2–specific multiplex PCR for nanopore sequencing (Appendix Table 2). We amplified cDNA (2.5 μL) in 2 multiplexed PCR reactions using Q5 Hot-Start DNA High-fidelity Polymerase (New England Biolabs, https://www.neb.com). We performed all consensus-level sequencing of SARS-CoV-2 using nanopore sequencing as described previously (*11*).

**Table 2:** Attack rates of coronavirus disease within dormitories and within roommates for dormitories with >10 cases, University of Wisconsin—Madison, Dane County, Wisconsin, USA, August 25–October 31, 2020*

Dormitory	No. residents	Residents with confirmed SARS-CoV-2 infection	Attack rates in roommates 2–14 d after index case†
Dormitory A	1,195	291/1,195 (24.4)	41/165 (24.8)
Dormitory B	924	295/924 (31.9)	32/172 (18.6)
Dormitory C	478	58/478 (12.1)	7/35 (20.0)
Dormitory D	181	19/181 (10.5)	2/9 (22.2)
Dormitory E	532	51/532 (9.6)	4/37 (10.8)
Dormitory F	384	31/384 (8.1)	5/23 (21.7)
Dormitory G	372	27/372 (7.3)	2/15 (13.3)
Dormitory H	319	20/319 (6.3)	1/14 (7.1)
Dormitory I	435	13/435 (3.0)	2/11 (18.2)
All other dormitories‡	1,342	51/1,342 (3.8)	5/33 (15.2)
Total†	6,162	856/6,162 (13.9)	101/514 (19.6)

*Values are no. positive/no. in category (%). †One room included in the roommate attack rate analysis housed 3 residents, whereas all others housed 2 residents; in the room with 2 susceptible residents, neither tested positive within 2–14 d of the index case. ‡Includes aggregated data from 10 dormitories not listed here that had <10 total cases each; attack rates in these halls were 1.9%–5.6%.

### Phylogenetic Analysis

We processed sequencing data using the ARTIC bioinformatics pipeline (https://github.com/artic-network/artic-ncov2019) scaled up for on-campus computing cores. The entire nanopore analysis pipeline is available at https://github.com/gagekmoreno/SARS-CoV-2-in-Southern-Wisconsin. We used all available full-length sequences from Dane County through January 31, 2021, for phylogenetic analysis using the tools implemented in Nextstrain custom builds (https://github.com/nextstrain/ncov) (*24**,**25*). We included 262 samples from students in dormitories A and B and 875 samples from persons tested at University of Wisconsin Hospital and Clinics (UWHC) from September 1, 2020–January 31, 2021; these samples represented ≈3% of all cases within Dane County, where UW-Madison is located, during this period. Persons using UWHC testing services included community members receiving preoperative testing, employees, inpatient and emergency department patients, patients from associated hospitals, and persons with known exposures. Of the 875 UWHC samples sequenced, 714 were collected on or after September 23, 2020, when the quarantine of dormitories A and B ended. We used this convenience sample to assess strains circulating within the Dane County community following the UW-Madison outbreak. We built time-resolved and divergence phylogenetic trees using standard Nextstrain tools and scripts. We filtered and cleaned metadata using custom Python (version 3.8; https://www.anaconda.com) scripts.

### Analyses Comparing Roommate Sequences

To test the hypothesis that roommate pairs are more likely to have similar viral sequences than nonroommate pairs, we linked data from 33 roommate pairs in which both roommates had sequencing data and performed a permutation test comparing the percent overlap in single-nucleotide polymorphism (SNP) identities between roommate pairs and random pairs of sequences derived from dormitories A and B. We performed a Mann-Whitney U test to compare the amount of diversity shared in roommate pairs and random pairs.

## Results

### Demographics, Symptom Manifestation, and Measures to Reduce Transmission

**Figure 1:**
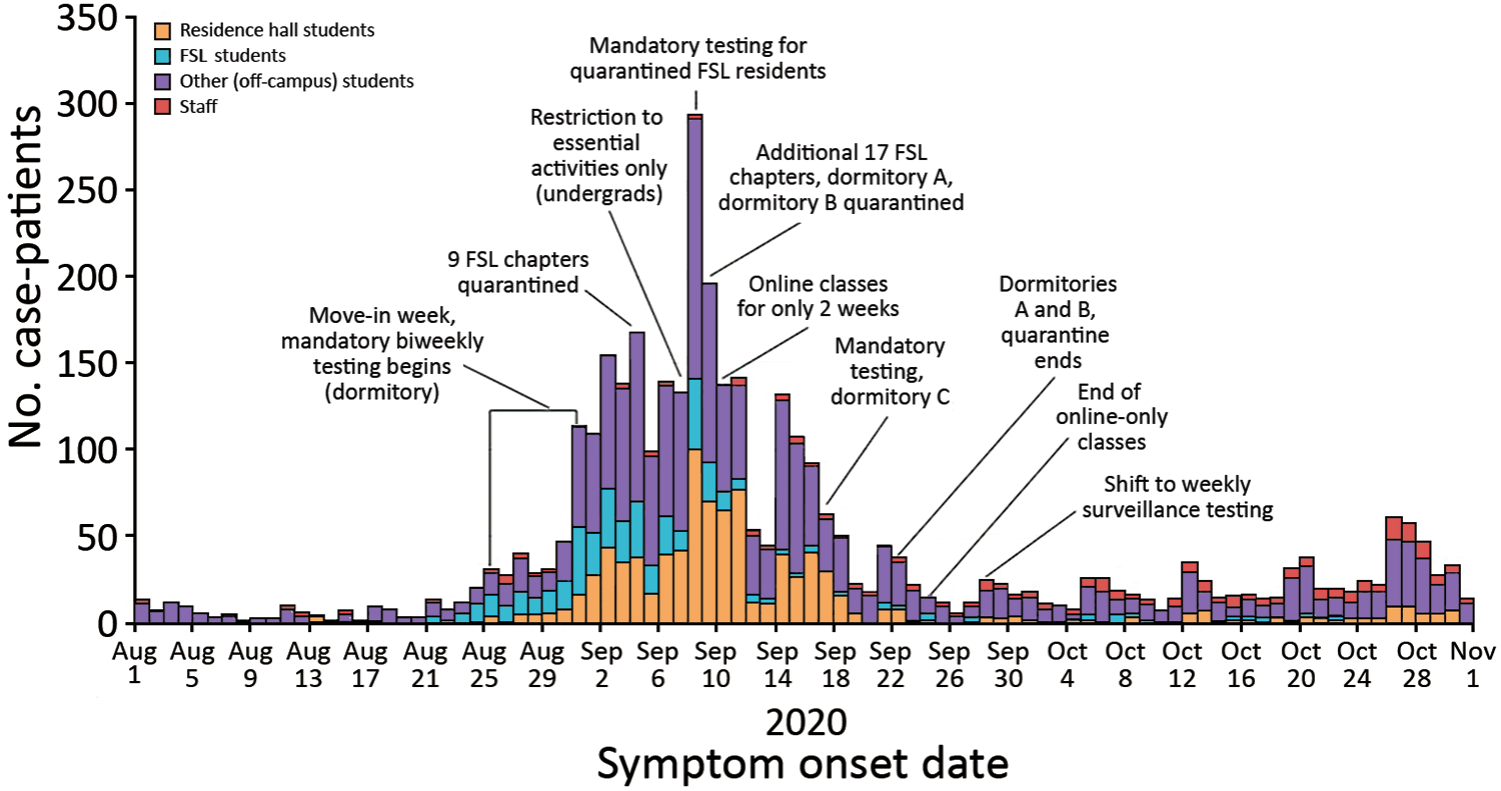
Overall epidemic curves of coronavirus disease cases among University of Wisconsin–Madison students and staff, Dane County, Wisconsin, USA, August 1–October 31, 2020. We categorized 10 student case-patients affiliated with both a dormitory and FSL as dormitory students. Student was considered the primary affiliation, such that any student who was also a staff member was categorized as a student. FSL, fraternity and sorority life.

During August 1–October 31, 2020, a total of 3,485 students and 245 staff affiliated with UW-Madison tested positive for SARS-CoV-2 by rRT-PCR, out of ≈45,540 enrolled students and 23,917 staff (Table 1). Cases in fraternity and sorority life (FSL) housing and other off-campus housing began rising before dormitory move-in week. UW-Madison–associated cases peaked during the week of September 6–12, 2020; soon after, cases began declining, showing a sustained decline through September and consistently low case counts in October (Figure 1). Most student (81.4%) and staff (80.4%) case-patients reported >1 symptom of COVID-19; 68.0% of students and 72.7% of staff met the Council of State and Territorial Epidemiologists clinical criteria for a COVID-19 case (Table 1) (*26*). Hospitalization was rare for both students and staff (<1.0%). Specimen collection occurred before symptom onset for 4.6% of student cases, whereas a positive result was reported before symptom onset for 0.7% of student cases. Among student case-patients, 902 (25.9%) were associated with an on-campus dormitory, 1,019 (29.2%) were associated with off-campus housing clusters, and 460 (13.2%) were associated with FSL (Table 1); the remainder were not linked to housing-specific clusters.

**Table 1:** Characteristics of University of Wisconsin-Madison student and staff coronavirus disease cases, Dane County, Wisconsin, USA, August 1–October 31, 2020*

Characteristic	Students, n = 3,485	Staff, n = 245
Mean age, y (range)	19.8 (17–72)	40.0 (20–88)
Sex		
M	1,677 (48.1)	114 (46.5)
F	1,807 (51.9)	131 (53.5)
Cluster affiliation†		
Dormitories	902 (25.9)	NA
Fraternity and sorority life	460 (13.2)	NA
Off-campus apartment	1,019 (29.2)	NA
No known affiliation with cluster	1,134 (32.5)	NA
Hospitalized		
Yes	4 (0.1)	1 (0.4)
No/unknown‡	3,481 (99.9)	244 (99.6)
Presence of symptoms§		
Symptomatic	2,838 (81.4)	197 (80.4)
Asymptomatic	647 (18.6)	48 (19.6)
Symptoms		
Headache	1,562 (44.8)	132 (53.9)
Sore throat	1,454 (41.7)	81 (33.1)
Fatigue	1,417 (40.7)	106 (43.3)
Cough	1,311 (37.6)	116 (47.4)
Runny nose	1,122 (32.2)	80 (32.7)
Muscle ache	1,021 (29.3)	100 (40.8)
Fever	918 (26.3)	75 (30.6)
Loss of smell	879 (25.2)	63 (25.7)
Loss of taste	777 (22.3)	53 (21.6)
Chills	822 (23.6)	56 (22.9)
Shortness of breath	336 (9.6)	19 (7.8)
Nausea	286 (8.2)	23 (9.4)
Diarrhea	247 (7.1)	19 (7.8)
Abdominal pain	126 (3.6)	12 (4.9)
Vomiting	43 (1.2)	7 (2.9)
Meets CSTE clinical criteria¶		
Yes	2,371 (68.0)	178 (72.7)
No	1,114 (32.0)	67 (27.3)
Timing of specimen collection relative to symptom onset		
Specimen collected on or after symptom onset date	2,275 (65.3)	162 (66.1)
Specimen collected before symptom onset date	162 (4.6)	7 (2.9)
No symptoms reported	647 (18.6)	48 (19.6)
Symptomatic, missing symptom onset date	401 (11.5)	28 (11.4)
Timing of positive report relative to symptom onset		
Positive reported on or after symptom onset date	2,411 (69.2)	167 (68.2)
Positive reported before symptom onset date	26 (0.7)	2 (0.8)
No symptoms reported	647 (18.6)	48 (19.6)
Symptomatic, missing symptom onset date	401 (11.5)	28 (11.4)

*Values are no. (%) except as indicated. Student affiliation was prioritized over staff, such that those identified as both students and staff are categorized as students. NA, not applicable. †Cluster affiliation categories are not mutually exclusive. ‡Cannot distinguish between no and unknown; there is only 1 checkbox in which hospitalization can be selected. §Anyone with >1 symptom is considered symptomatic; asymptomatic does not distinguish between those who were truly asymptomatic and those who were missing symptom information. ¶CSTE clinical criteria are met if the case-patient has either cough or shortness of breath, or >2 of the following symptoms: fever, chills, myalgia, headache, sore throat, loss of smell, or loss of taste.

Multiple mitigation measures were put into place to reduce transmission during September 6–12, 2020. Those measures included suspending in-person classes and events, prohibiting nonsanctioned social activities, holding additional mass testing events, and quarantining all students in dormitories A and B during September 9–23, 2020 (Figure 1). The local health department also required testing and quarantine for 26 FSL house chapters.

### Infections among Students in Dormitories

**Figure 2:**
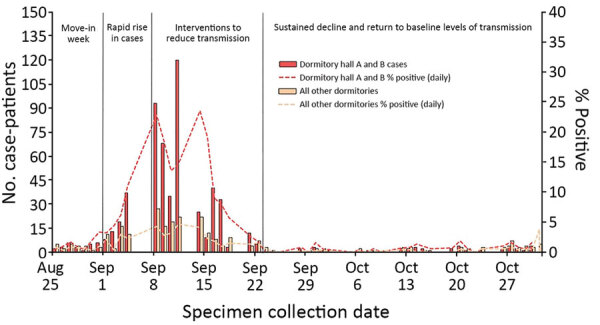
Coronavirus disease epidemic curves and percent positivity for University of Wisconsin–Madison students living in dormitories A and B versus all other dormitories, Dane County, Wisconsin, USA, August 25–October 31, 2020.

Across all dormitories, 5,820/6,162 students (94.4%) were tested during move-in week (August 25–31, 2020); mean turnaround time from test to result was 2 days (interquartile range 1–2 days). Thirty-four students (0.6%) tested positive at move-in without documentation of a previous positive test in the previous 90 days; these students were moved into isolation dorms. Overall, 856/6,162 (13.9%) students living in the 19 on-campus dormitories had a positive SARS-CoV-2 specimen collected through campus testing during August 25–October 31, 2020; attack rates in dormitories were 1.9%–31.9% (Table 2**)** during this time. Fifteen dormitories had attack rates of <10.0%, 2 had attack rates of 10.0%–20.0%, and 2 had attack rates >20.0%. Dormitories A and B accounted for 68.5% of all dormitory cases (586/856), but only 34.4% of all students living in dormitories (2,119/6,162) (Figure 2).

**Figure 3:**
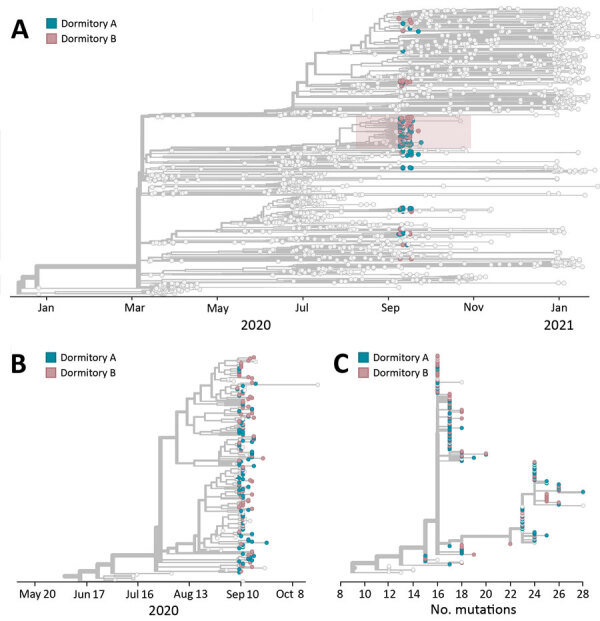
Phylogenetic tree of the coronavirus disease outbreak in dormitories A and B, University of Wisconsin–Madison, Dane County, Wisconsin, USA, January 2020–January 2021. A) Phylogenetic tree of all cases sequenced in Dane County, Wisconsin (light gray tips) during January 2020–January 2021 and cases sequenced in each dormitory. Pink shading indicates cluster associated with dormitories A and B. B) Expanded view of phylogenetic tree of the large cluster of cases associated with dormitories A and B during the September 2020 outbreak. C) Mutations relative to the initially identified severe acute respiratory syndrome coronavirus 2 genome in Wuhan, China (GenBank accession no. MN908947.3), during the outbreak in dormitories A and B.

In addition, we used a divergence phylogeny, colored by dormitory, to compare the number of mutations present in each sequence relative to the initial SARS-CoV-2 reference virus (GenBank accession no. MN908947.3). If dormitories A and B had distinct but contemporaneous outbreaks, we might expect viral sequences from the 2 halls to segregate into distinct taxa on a divergence tree. However, the tree illustrates that substantial mixing of viral genetic lineages between the dormitories occurred, indicating that outbreaks of COVID-19 within these dormitories were not distinct and resulted from intermingling between residents (Figure 3, panel C). 

### Whole-Genome Sequencing among Student Samples from Dormitories A and B

We sequenced complete viral genomes from 262 (44.7%) of 586 specimens from students living in dormitories A and B (Figure 3). Using a Dane County–centric phylogeny, we visualized the relationship of SARS-CoV-2 viruses circulating in dormitories A and B (Figure 3). Almost two thirds of sequences from the dormitories (172/262; 65.6%) formed a cluster in the 20A clade (PANGO lineage B.1.369) (Figure 3, panel B). This cluster contains a unique spike mutation encoding a glutamic acid-to-glutamine substitution at spike residue 780 (S E780Q), which was not seen in Dane County before this outbreak. This mutation was not subsequently found in 467 sequenced specimens from Dane County (of 15,740 positive tests, a sequencing coverage of 2.96%) during November 11, 2020–January 31, 2021.

**Figure 4:**
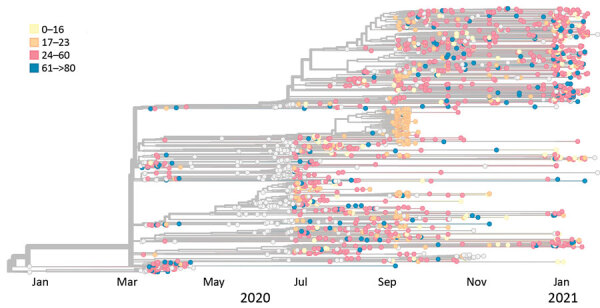
Phylogenetic tree of severe acute respiratory syndrome coronavirus 2 specimens sequenced in Dane County, Wisconsin, USA, January 2020–January 2021, coded by age of case-patient providing specimen.

The remaining 90 dormitory sequences clustered with the 20A (32/262), 20G (30/262), 20C (24/262), and 20B (4/262) clades. Sequences clustering in those remaining clades were more closely related to viral lineages concurrently circulating in Dane County, suggesting these persons became infected in the community. During September 23, 2020–January 31, 2021, a total of 75.3% (538/714) of new sequences in Dane County were classified as 20G clade, 15.1% (108/714) as 20A clade, 7.0% (50/714) as 20C clade, and 2.5% (18/714) as 20B clade. The large cluster in dormitories A and B was almost exclusively among case-patients 17–23 years of age (Figure 4).

### Risk for Transmission between Roommates

Across all dormitories, 81.6% of residents had a roommate. Percentage positivity was higher overall among students with roommates (15.4%) than those without roommates (7.3%) (p<0.0001). Of the 514 students who had a roommate test positive but had not yet tested positive themselves, 101 (19.6%) tested positive within 2–14 days. (Table 2). Genetic distance comparisons between roommate pairs and nonroommate pairs within dormitories A and B revealed significantly higher levels of overlap in SNV identities between roommate pairs compared to random pairs. Specifically, 32/33 (97.0%) roommate pairs had viruses that contained 100.0% identical consensus sequences, whereas identical consensus sequences were found in 1,062/33,930 (3.1%) of randomly assigned pairs (p<0.0001).

## Discussion

An outbreak of COVID-19 occurred at UW-Madison at the beginning of the fall semester. Over the course of our investigation, ≈14.0% of students living in dormitories tested positive; those living with roommates were more likely to test positive. Shortly after the UW-Madison outbreak began, mitigation measures were rapidly implemented, and a rapid decline in cases was observed. Ninety residence-hall sequences clustered with viruses circulating in Dane County, suggesting mixing between the university and Dane County. However, we did not detect evidence of transmission of the predominant viral lineages associated with dormitories A or B beyond these dormitories within Dane County in a convenience sample of sequenced specimens collected in the months following the outbreak.

Testing at the time students moved into dormitories identified some introductions of SARS-CoV-2 onto campus, and UW-Madison isolated infected students. However, the average 2-day turnaround time for test results meant transmission might have occurred while students were awaiting their results. Therefore, when implementing move-in testing, quarantining students until results have been received may help prevent transmission among asymptomatic students awaiting results (*27*). Move-in testing also may fail to identify students who have recently been infected and do not yet have detectable levels of SARS-CoV-2 virus (*28*) and cannot prevent new infections if the virus is already circulating in the community. Our results suggest the importance of supplementing move-in testing with ongoing serial testing and additional mitigation steps to effectively prevent ongoing transmission and community spread.

UW-Madison conducted biweekly serial screening testing for students in dormitories with relatively short turnaround time (mean 2 days), enabling the university to identify and isolate students with SARS-CoV-2 infections, quarantine roommates, and conduct contact tracing. Still, more frequent testing may have enabled more rapid case detection and initiation of isolation and quarantine procedures, preventing further transmission. A modeling study of COVID-19 spread within IHEs suggested that testing every 2 days would be needed to control the spread of SARS-CoV-2 (*29*). Recognizing this potential for rapid spread, UW-Madison increased the frequency of testing to twice per week for students living on-campus and off-campus in nearby ZIP codes and reduced turnaround time for results to <24 hours for the spring 2021 semester (*30**,**31*). Further evaluation of serial testing strategies is needed to determine optimal testing frequency in IHE settings and to prioritize populations for testing when capacity is limited. The high proportion of infected students who were symptomatic (>80.0%) suggests that, even in young adults, SARS-CoV-2 infection is frequently associated with at least mild symptoms, reinforcing the importance of educating students on COVID-19 symptoms, symptom monitoring, testing, and self-isolation when even mild symptoms develop (*32*).

Roommates live in close contact with each other, providing substantial opportunities for transmission (*32*). At UW-Madison, roommates were not required to wear masks within their rooms because this measure was considered impractical and unenforceable. Roommates of confirmed case-patients within dormitories had an estimated attack rate of 19.6%, and a larger proportion of students with roommates tested positive over the investigation period than those without. Furthermore, SARS-CoV-2 genomes collected from 33 roommate pairs found a high proportion of identical sequences, suggesting transmission occurred either within the roommate pair or from a shared exposure. Given the elevated risk for infection associated with having a roommate, efforts to reduce the density of dormitories, including single-occupancy rooms when available, may reduce transmission (*1*).

Two dormitories accounted for more than two thirds of all confirmed cases among students living in dormitories during the investigation period, although these 2 halls accounted for only one third of students living in on-campus housing. Transmission may have occurred within the dormitories but may have also occurred in other undetected settings (e.g., bars, private residences, fraternities, or sororities) that residents of dormitories A and B might have visited more frequently than did students living in other dormitories (*33**,**34*). The sequencing data strongly suggest that the clusters in dormitories A and B, which are located close to each other and share dining and recreation spaces, were not independent and were the result of intermingling. Viral genome sequencing is an important tool in understanding the transmission dynamics between UW-Madison students and the broader community (*11*–*16*; C.S. Richmond et al., unpub. data; M. Zeller et al., unpub. data). Our sequencing data covering 44.7% of student case-patients living in dormitories A and B, 7.5% of all student case-patients, and 3.0% of community samples from Dane County did not find evidence that viruses from this cluster subsequently circulated at high frequencies in the community.

The first limitation of our analysis is that full lists of off-campus students and staff and their COVID-19 testing histories were not available; therefore, attack rates could be calculated only for students living in on-campus dormitories. We did not examine data related to race, ethnicity, and other social determinants of health. Occupancy levels remained fluid throughout the semester, but available data used for dormitory census calculations represented a single point in time at the end of the outbreak, when occupancy was lower than at the start of the semester. UW-Madison’s rapid implementation of multiple interventions limits our ability to determine the effectiveness of individual interventions. Specimens from students living in dormitories A and B were targeted for sequencing to understand transmission patterns within and across these housing units. Therefore, our sequencing results should not be generalized to the campus at large; transmission events may have occurred after campus-related clusters outside of dormitories A and B. Other studies assessing trends in cases over time have suggested that university outbreaks preceding broader community outbreaks may be caused by transmission from universities to community members, a possibility that we cannot rule out (*10*). In addition, sequencing of Dane County specimens in Nextstrain represented a small proportion of the total number infections within the county (≈3.0%) and were sampled nonrandomly among clients of a large testing provider. Therefore, it is possible that descendant infections from dormitory A and B clusters occurred in Dane County but were not captured in the convenience sample from the community.

This investigation described an outbreak in which COVID-19 spread rapidly among university students at UW-Madison. Given the swift rise in cases, being able to quickly identify outbreaks and rapidly implement mitigation strategies by a coordinated universitywide response in collaboration with public health authorities is critical in halting transmission. Large-scale quarantines in congregate living situations (e.g., dormitories) and suspension of on-campus activities may be effective during large-scale outbreaks, if put in place rapidly and effectively. This investigation demonstrates using genomic surveillance to provide a more comprehensive understanding of transmission dynamics both in specific outbreak settings and in the general population. These tools can be used by universities and health departments to monitor spillover into the community and inform campus and community mitigation efforts.

## References

[1] Wilson E, Donovan CV, Campbell M, Chai T, Pittman K, Seña AC, et al. Multiple COVID-19 clusters on a university campus—North Carolina, August 2020. MMWR Morb Mortal Wkly Rep. 2020;69:1416–8. 10.15585/mmwr.mm6939e3 33001871 PMC7537562

[2] Fox MD, Bailey DC, Seamon MD, Miranda ML. Response to a COVID-19 outbreak on a university campus—Indiana, August 2020. MMWR Morb Mortal Wkly Rep. 2021;70:118–22. 10.15585/mmwr.mm7004a3 33507894 PMC7842813

[3] Salvatore PP, Sula E, Coyle JP, Caruso E, Smith AR, Levine RS, et al. Recent increase in COVID-19 cases reported among adults aged 18–22 years—United States, May 31–September 5, 2020. MMWR Morb Mortal Wkly Rep. 2020;69:1419–24. 10.15585/mmwr.mm6939e4 33006586 PMC7537557

[4] Cai W, Ivory D, Semple K, Smith M, Lemonides A, Higgins L, et al. Tracking coronavirus cases at U.S. colleges and universities. The New York Times. 2021 [cited 2021 Mar 1]. https://www.nytimes.com/interactive/2021/us/college-covid-tracker.html

[5] Centers for Disease Control and Prevention. COVID-19 parental resources kit—young adulthood. Updated December 28, 2020 [cited 2021 Apr 15]. https://www.cdc.gov/coronavirus/2019-ncov/daily-life-coping/parental-resource-kit/young-adulthood.html

[6] Bialek S, Boundy E, Bowen V, Chow N, Cohn A, Dowling N, et al.; CDC COVID-19 Response Team. Severe outcomes among patients with coronavirus disease 2019 (COVID-19)—United States, February 12–March 16, 2020. MMWR Morb Mortal Wkly Rep. 2020;69:343–6. 10.15585/mmwr.mm6912e2 32214079 PMC7725513

[7] Andrews JL, Foulkes L, Blakemore SJ. Peer influence in adolescence: public-health implications for COVID-19. Trends Cogn Sci. 2020;24:585–7. 10.1016/j.tics.2020.05.001 32444200 PMC7205648

[8] Walke HT, Honein MA, Redfield RR. Preventing and responding to COVID-19 on college campuses. JAMA. 2020;324:1727–8. 10.1001/jama.2020.20027 32991681 PMC9648565

[9] Leidner AJ, Barry V, Bowen VB, Silver R, Musial T, Kang GJ, et al. Opening of large institutions of higher education and county-level COVID-19 incidence—United States, July 6–September 17, 2020. MMWR Morb Mortal Wkly Rep. 2021;70:14–9. 10.15585/mmwr.mm7001a4 33411699 PMC7790156

[10] Pray IW, Kocharian A, Mason J, Westergaard R, Meiman J. Trends in outbreak-associated cases of COVID-19—Wisconsin, March–November 2020. MMWR Morb Mortal Wkly Rep. 2021;70:114–7. 10.15585/mmwr.mm7004a2 33507887 PMC7842809

[11] Moreno GK, Braun KM, Riemersma KK, Martin MA, Halfmann PJ, Crooks CM, et al. Revealing fine-scale spatiotemporal differences in SARS-CoV-2 introduction and spread. Nat Commun. 2020;11:5558. 10.1038/s41467-020-19346-z 33144575 PMC7609670

[12] Fauver JR, Petrone ME, Hodcroft EB, Shioda K, Ehrlich HY, Watts AG, et al. Coast-to-coast spread of SARS-CoV-2 during the early epidemic in the United States. Cell. 2020;181:990–996.e5. 10.1016/j.cell.2020.04.021 32386545 PMC7204677

[13] Lemieux JE, Siddle KJ, Shaw BM, Loreth C, Schaffner SF, Gladden-Young A, et al. Phylogenetic analysis of SARS-CoV-2 in Boston highlights the impact of superspreading events. Science. 2021;371:eabe3261. 10.1126/science.abe3261 33303686 PMC7857412

[14] Miller D, Martin MA, Harel N, Tirosh O, Kustin T, Meir M, et al. Full genome viral sequences inform patterns of SARS-CoV-2 spread into and within Israel. Nat Commun. 2020;11:5518. 10.1038/s41467-020-19248-0 33139704 PMC7606475

[15] Maurano MT, Ramaswami S, Zappile P, Dimartino D, Boytard L, Ribeiro-Dos-Santos AM, et al. Sequencing identifies multiple early introductions of SARS-CoV-2 to the New York City region. Genome Res. 2020;30:1781–8. 10.1101/gr.266676.120 33093069 PMC7706732

[16] Bedford T, Greninger AL, Roychoudhury P, Starita LM, Famulare M, Huang ML, et al.; Seattle Flu Study Investigators. Cryptic transmission of SARS-CoV-2 in Washington state. Science. 2020;370:571–5. 10.1126/science.abc0523 32913002 PMC7810035

[17] University of Wisconsin—Madison. At a glance factsheet. 2021 [cited 2021 Mar 22]. https://www.wisc.edu/pdfs/uwmadison-factsheet-jan-2021.pdf

[18] University of Wisconsin—Madison. COVID-19 response: Badger pledge for students. Madison (WI): The University; 2020.

[19] Centers for Disease Control and Prevention. Interim guidance on duration of isolation and precautions for adults with COVID-19. February 13, 2021 [cited 2021 Mar 31]. https://www.cdc.gov/coronavirus/2019-ncov/hcp/duration-isolation.html

[20] Public Health Madison & Dane County. Emergency order #8. July 7, 2020 [cited 2021 Mar 1]. https://publichealthmdc.com/documents/2020-07-07_Order_8.pdf

[21] Quick J, Grubaugh ND, Pullan ST, Claro IM, Smith AD, Gangavarapu K, et al. Multiplex PCR method for MinION and Illumina sequencing of Zika and other virus genomes directly from clinical samples. Nat Protoc. 2017;12:1261–76. 10.1038/nprot.2017.066 28538739 PMC5902022

[22] Quick J. nCoV-2019 sequencing protocol v3 (LoCost) version 3. August 25, 2020 [cited 2021 Mar 9]. https://www.protocols.io/view/ncov-2019-sequencing-protocol-v3-locost-bh42j8ye

[23] Quick J. nCoV-2019 sequencing protocol v2 (GunIt) version 2. April 9, 2020 [cited 2021 Mar 9]. https://www.protocols.io/view/ncov-2019-sequencing-protocol-v2-bdp7i5rn?version_warning=no

[24] Hadfield J, Megill C, Bell SM, Huddleston J, Potter B, Callender C, et al. Nextstrain: real-time tracking of pathogen evolution. Bioinformatics. 2018;34:4121–3. 10.1093/bioinformatics/bty407 29790939 PMC6247931

[25] Sagulenko P, Puller V, Neher RA. TreeTime: Maximum-likelihood phylodynamic analysis. Virus Evol. 2018;4:vex042. 10.1093/ve/vex042 29340210 PMC5758920

[26] Centers for Disease Control and Prevention. Coronavirus disease 2019 (COVID-19) 2020 interim case definition, approved August 5, 2020. CSTE position statement: interim-20-ID-02 [cited 2021 Jan 7]. https://ndc.services.cdc.gov/case-definitions/coronavirus-disease-2019-2020-08-05

[27] Yamey G, Walensky RP. Covid-19: re-opening universities is high risk. BMJ. 2020;370:m3365. 10.1136/bmj.m3365 32873595

[28] Centers for Disease Control and Prevention. COVID-19 testing overview. 2021 [cited 2021 Feb 23]. https://www.cdc.gov/coronavirus/2019-ncov/symptoms-testing/testing.html

[29] Paltiel AD, Zheng A, Walensky RP. Assessment of SARS-CoV-2 screening strategies to permit the safe reopening of college campuses in the United States. JAMA Netw Open. 2020;3:e2016818. 10.1001/jamanetworkopen.2020.16818 32735339 PMC7395236

[30] University of Wisconsin. What is the testing process for undergraduates living in residence halls for spring semester? Madison (WI): The University; 2021.

[31] University of Wisconsin. What is the testing process for undergraduates living off campus for spring semester? Madison (WI): The University; 2021

[32] Centers for Disease Control and Prevention. COVID-19 guidance for shared or congregate housing. Last updated December 31, 2020 [cited 2021 Feb 23]. https://www.cdc.gov/coronavirus/2019-ncov/community/shared-congregate-house/guidance-shared-congregate-housing.html

[33] Harris JE. Geospatial analysis of the September 2020 coronavirus outbreak at the University of Wisconsin—Madison: did a cluster of local bars play a critical role? National Bureau of Economic Research Working Paper 28132. 2020. [cited 2021 Aug 31]. https://www.nber.org/papers/w28132.

[34] Vang KE, Krow-Lucal ER, James AE, Cima MJ, Kothari A, Zohoori N, et al. Participation in fraternity and sorority activities and the spread of COVID-19 among residential university communities—Arkansas, August 21–September 5, 2020. MMWR Morb Mortal Wkly Rep. 2021;70:20–3. 10.15585/mmwr.mm7001a5 33411698 PMC7790151

